# Fecal Proteomic Analysis in Healthy Dogs and in Dogs Suffering from Food Responsive Diarrhea

**DOI:** 10.1155/2019/2742401

**Published:** 2019-01-03

**Authors:** Matteo Cerquetella, Giacomo Rossi, Andrea Spaterna, Beniamino Tesei, Alessandra Gavazza, Graziano Pengo, Stefania Pucciarelli, Luca Scortichini, Gianni Sagratini, Massimo Ricciutelli, Andrea Marchegiani, Silvia Vincenzetti

**Affiliations:** ^1^School of Biosciences and Veterinary Medicine, University of Camerino, Via Circonvallazione 93/95, 62024 Matelica (MC), Italy; ^2^S. Antonio Clinic, Madignano, S.S 415 Paullese 6, 26020 Madignano (CR), Italy; ^3^School of Pharmacy, University of Camerino, Via Sant'Agostino 1, 62032 Camerino (MC), Italy

## Abstract

Different laboratory markers are routinely used in the diagnosis and management of gastrointestinal (GI) disease in dogs. In the present study, starting from feces from both healthy dogs and dogs suffering from food responsive diarrhea (FRD), we tried to find proteins differently expressed in the two groups of dogs, by using a proteomic approach. Interestingly, we found that the immunoglobulin J-chain isoform 1 (species:* Canis lupus familiaris*) was identified only in diseased dogs (not in healthy). J-chain combines especially IgA monomers to IgA dimers and plays a crucial role for their secretions into mucosal interface. Being the first study of that kind in the dog, it is only possible to hypothesize that their presence could be likely due to an increased activation of the immune system or to a mucosal damage or both in FRD patients. Similarly, it is still impossible to assess whether this protein could be used as diagnostic/prognostic marker of GI disease; however, this study represents a promising first step toward fecal proteomics in canine GI disorders.

## 1. Introduction

Food responsive diarrhea (FRD) is included in the group of canine chronic enteropathies (CCE) [1] and is considered as the presence of a gastrointestinal (GI) disease lasting from more than 3 weeks that clinically improve after the administration of specific diets (elimination diet) or of diets containing hydrolyzed proteins [2]. Normally, the diagnosis is made after a positive response to the dietary trial, and even if some specific fecal markers have been investigated in dogs suffering from GI disease (e.g., fecal *α*1-proteinase inhibitor, N-methylhistamine, fecal calprotectin, S100A12, etc.) [3–7], a fecal proteomic study has never been performed on canine FRD patients.

Proteomics is the comprehensive study of the proteome (proteins' structure, functions, etc.) of a specific environment, and it is one strategy in a wider “-omic” approach [8, 9]. In canine medicine, proteomics has been applied to different biological fluids like serum, urine, cerebrospinal fluid, bronchoalveolar lavage fluid, ovarian follicular fluid, tears, etc. [10, 11] and to tissues such as mammary cells, muscles, liver, etc. [11]. Proteomic analyses have also been performed in some pathological conditions of the dog such as tumors (e.g., mammary gland, cutaneous mast cell tumors, lymphoma, and prostate), muscular dystrophy, lethal acrodermatitis, babesiosis, mitral valve disease, obesity-related metabolic dysfunction, reduced renal function and tubulointerstitial fibrosis, etc. [11–21].

The aim of the present study was to detect and identify, by a proteomic approach, for the first time in the dog, most represented proteins in feces from healthy dogs of different breeds and in dogs suffering from food responsive diarrhea and then to compare results between the two groups of dogs.

## 2. Materials and Methods

### 2.1. Dogs

We investigated the fecal samples of 7 healthy dogs and of 12 dogs suffering from FRD. Inclusion criteria for healthy dogs were absence of GI signs or of any other clinical sign and absence of concomitant diseases + no pre/probiotic administration + no change in diet, within the last 3 months (they were all periodically controlled as included in a volunteer blood donor program). Among the dogs included in the control group, there were 2 Golden Retrievers, 1 Dobermann, 1 German Shepherd, 1 mestizo, 1 Pit Bull, and 1 Weimaraner. Five were females and two males, and the mean age was 7.5 years. Dogs included in the FRD group were presenting GI signs from more than 3 weeks, and all responded to dietary changes. Routine laboratory and instrumental (ultrasonography and radiology) evaluations were consistent with diagnosis of FRD in all dogs. Histopathology performed on endoscopic biopsy samples was, in a major part of enrolled cases, consistent with lymphocytic-plasmacytic enteritis (LPE) forms. This histological condition is not specific but concordant with FRD; indeed food responsive enteropathy and steroid-responsive enteropathy or inflammatory bowel disease (IBD) cannot be discriminated based on histopathological results. With regard to breeds, there were 2 mestizo, 1 Boxer, 1 Bull Terrier, 1 Cavalier King Charles spaniel, 1 French Bulldog, 1 Golden Retriever, 1 Labrador Retriever, 1 Maltese, 1 Shih-Tzu, 1 Siberian Husky, and 1 Staffordshire Bull Terrier. Seven were males and five females, and the mean age was 5.3 years. All dogs were regularly dewormed.

### 2.2. Fecal Samples Preparation and Protein Extraction

Naturally voided fecal samples were collected (with owners' informed consent) at the time of first diagnosis, immediately after production, and stored at -20°C. 2.0 grams of frozen faces from healthy group and from FRD group have been separately weighed and resuspended in 3 ml of phosphate buffered saline (PBS) containing a protease inhibitor cocktail (Sigma-Aldrich, Saint Louis, MO), diluted 1:100. Both samples were separately subjected to agitation through a magnetic stirrer for 30 min in ice. Subsequently the two mixtures were centrifuged at10000* x*g at 4°C. After centrifugation, the two supernatants (from healthy group and from FRD group) were collected, filtered three times with a filter paper, and one more time with a 0.22 *μ*m filter (Whatman, Maidstone, UK). These consecutive filtering steps were performed in order to eliminate contaminations of proteins deriving from gut microflora. To the obtained filtered samples, ammonium sulphate (Sigma-Aldrich, Saint Louis, MO) was slowly added to each sample to achieve saturation at 90%, in order to concentrate proteins. This operation was performed maintaining the samples on ice and under agitation with a magnetic stirrer. After 30 min incubation in ice, the samples were subsequently centrifuged at 10000xg for 30 min at 4°C. After centrifugation, the supernatants were discarded and each pellet was resuspended in 500*μ*l PBS. The two samples were then dialyzed by ultrafiltration membranes (MWCO 3 kDa, Spectra/Por®, Repligen Corporation, Waltham, MA). After dialysis the total protein content was determined by the Bradford method [22].

### 2.3. Two-Dimensional Polyacrylamide Gel Electrophoresis (2DE)

2DE experiments were performed in triplicate for each group of samples. Before 2DE, samples were processed as follows: 800 *μ*g of each total fecal protein group (from healthy group and from FRD group), extract as described in the previous section, was cleaned by the 2-D Clean-Up Kit (GE-Healthcare Life Sciences, Uppsala, Sweden) in order to eliminate contaminants, and then was dissolved in a 350 *μ*L of rehydration solution (8 M urea; 2% (w/v) 3-[(3-Cholamidopropyl) dimethylammonio]-1-propanesulfonate (CHAPS); 65 mM dithiothreitol (DTT); 0.001% (w/v) bromophenol blue; 0.5% (v/v) IPG buffer, pH range 3-10). The first dimension was performed at a pH range of 3-10 (Immobiline DryStrip, IPG-strip, length 18 cm, GE-Halthcare) on an IPGphor isoelectric focusing cell (GE-Healthcare) and run as previously described [23, 24]. The second dimension was performed by a 13% SDS-PAGE using a Protean II apparatus (Bio-Rad, Hercules, CA, USA) and run as previously described [23, 24]. At the end of the electrophoretic run, the gels were recovered, stained with 0.1% Coomassie Brilliant Blue R250, destained, and scanned at 600 dpi resolution. Image analysis was performed using the PDQuest software (Version 7.1.1; Bio-Rad Laboratories), according to the protocols provided by the manufacturer in order to define spot-intensity calibration, spot detection, background abstraction, calibration, and calculation of molecular mass and isoelectric point (pI) [23, 24]. The pIs were determined using a linear 3-10 distribution, and the molecular mass determination was based on the markers Bio-Rad low range (phosphorylase b, 97.4 kDa; bovine serum albumin, 66.2 kDa; ovalbumin 45.0 kDa; carbonic anhydrase, 31 kDa; soybean trypsin inhibitor, 21.5 kDa; lysozyme, 14.4 kDa). After PDQuest analysis, the spots were manually excised (1 mm in diameter) and the protein extracted from the gel following the protocol of Shevchenko and coworkers [25] and subsequently subjected to LC-MS/MS analysis.

### 2.4. Liquid Chromatography-Tandem Mass Spectrometry (LC-MS/MS) Analysis

After the digestion, the tryptic peptides were dissolved in 100 *μ*l of 0.1% (v/v) trifluoroacetic acid and subjected to a reversed phase chromatography (C18 Gemini-NX, *μ*l particle size, 110 Å pore size, 250x4.6 mm, Phenomenex, Torrance, CA.) connected to a HPLC Agilent Technologies 1100 Series (Agilent Technologies, Santa Clara, CA.). The column effluent was analyzed by MS using an electrospray ion trap mass spectrometer (Agilent Technologies LC/MSD Trap SL) operating in positive ion mode over the mass range 300-2200 amu (atomic mass units). MS spray voltage was 3.5 kV and the capillary temperature was maintained at 300°C. Obtained spectra were extracted and analyzed by the MASCOT software (www.matrixscience.com) and by the SONAR software (http://hs2.proteome.ca/prowl/knexus.html) with the following search parameters: database, NCBInr; taxonomy,* Eukaryota*; enzyme, trypsin; peptide tolerance, 1.2 Da; MS/MS tolerance, 0.6 Da and allowance of one missed cleavage [26].

### 2.5. Statistical Analysis

Data were analyzed by using GraphPadPrism® 6.01 software. One-way ANOVA with Tukey correction for multiple comparisons were employed when three or more groups were compared. Significant differences between means were indicated when P < 0.05.

## 3. Results and Discussion

Thanks to the extraction protocol described under the Materials and Methods section, it was possible to obtain a protein concentration of 2.68±0.27mg/ml starting from 2 grams of feces. It is important to underline the great availability of the starting material (feces), which compensates the low quantity of proteins that can be extracted from the feces through this procedure. Furthermore, another important consideration is that regardless of the consistency of the starting material, a fixed amount of total proteins (800 *μ*g) on the two-dimensional electrophoresis can be always loaded.

**Figure 1:**
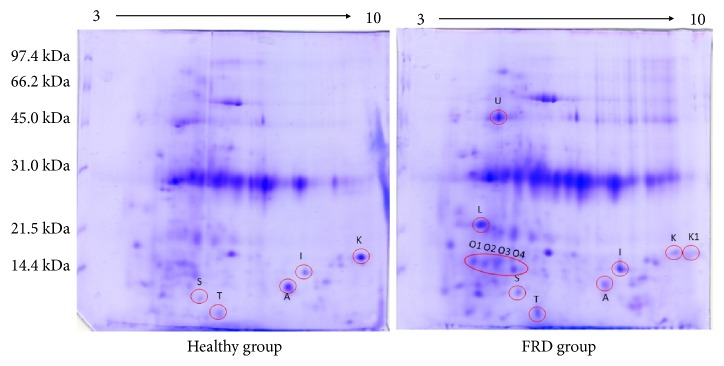
2DE map of the feces proteins from healthy dog group and FRD dog group. The experiments were performed in triplicate for the two samples. Differently expressed protein spots are evidenced in red (see also Table 1 for protein identification). First dimension has been performed using an immobilized pH 3–10 linear gradient strip whereas the second dimension was a 13% SDS-PAGE. The standards were Bio-Rad low molecular weight (phosphorylase b, 97.4 kDa; bovine serum albumin, 66.2 kDa; ovalbumin 45.0 kDa; carbonic anhydrase, 31.0 kDa; soybean trypsin inhibitor, 21.5 kDa; lysozyme, 14.4 kDa).

**Figure 2:**
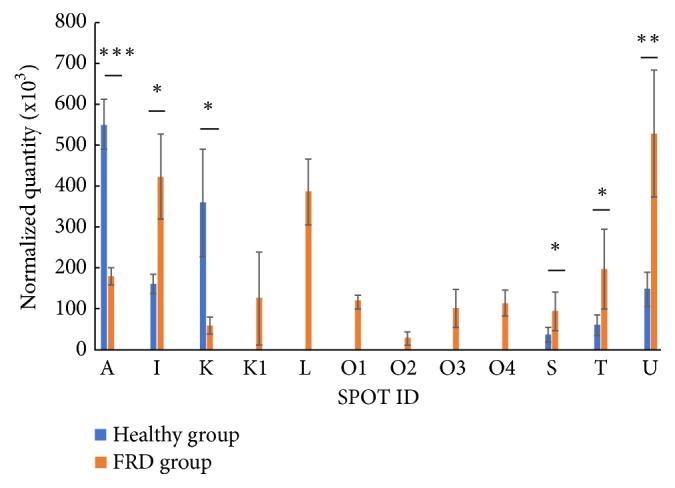
Quantitative analysis of each spot in the healthy group and in the FRD group. Data are shown as mean values ± SE. ^∗∗∗^ P<0.005; ^∗∗^P<0.01; ^∗^P<0.05.

The protein expression profile of fecal samples of healthy dogs and of dogs suffering from FRD was examined by 2DE in the pH range 3-10.  Figure 1 shows a representation of the protein spots comparison between the two samples. PDQuest analysis revealed the presence of 12 spots differentially expressed in the fecal samples of healthy subjects and in the subjects affected to FRD. Among them, the presence of the spots K1, L, O1, O2, O3 and O4 were found only in the feces of dogs suffering from FRD. In Figure 2 is shown the normalized quantity of each spot, whereas the experimental pI values and the molecular weights of the spot proteins, compared with the theoretical values found by the MASCOT or SONAR software, are shown in Table 1. Before to perform the spots identification, by LC-MS/MS in combination with a databases search, the twelve spots of interest were excised from 2-D gels and digested with trypsin. The obtained results are shown in Table 1. The spots present mainly or only in the feces of healthy dogs are: spot A (Hemoglobin subunit beta,* Bos Taurus*) and spot K (Putative Cytochrome P450,* Oryza sativa*). The spots found mainly in the feces of dogs affected to FRD are: spot I (Hypothetical protein,* Streptomyces sp.*); spots S and T (DTW domain-containing protein,* Microbulbifer donghaiensis* and hypothetical protein U973_01647,* Staphylococcus aureus*, respectively). The spots K1 and L are found only in feces of FRD dogs and correspond respectively to UDP-N-acetylglucosamine diphosphorylase (*Zea mays*) and to isopentenyl-diphosphate delta-isomerase (*Streptomyces sp.*). The spot O2 has been identified as the immunoglobulin J-chain isoform 1 (*Canis lupus familiaris*), whereas spot O1, O3 and O4 were not identified. The spot U corresponds to the coproporphyrinogen III oxidase (*Pontibacillus chungwhensis*).

Fecal proteome has been investigated in human medicine [27, 28], but to the authors knowledge this is the first time that it is studied in canine medicine, in patients suffering from FRD. In human medicine, the study of fecal proteome is considered to have a potential on diseases like Crohn's disease, irritable bowel syndrome, colorectal cancer, etc. [28], and proteomics, more in general, has been applied with interesting results and promising perspectives in men suffering from food allergy [9].

Analyzing proteins from canine feces, the most interesting spot we found was the spot O2, identified as immunoglobulin J-chain isoform 1. The joining (J) chain is expressed in several tissues, most abundant in gastrointestinal tract and lymphoid tissues. J-chain combines IgM (pentameric IgM) and (especially) IgA monomers into the dimeric IgA molecule (but larger polymers are also possible) and it is extremely important for its transport across the epithelium into mucosal secretions, mediated by the polymeric immunoglobulin mediated receptor (pIgR) [29–32]. pIgR mediates the active transport of bound polymeric Ig from the basolateral to the apical face of the exocrine epithelial cells. Therefore J-chain has an important role in the releasing of secretory antibodies to the mucosal interface [30]. Thus, it appears that the J-chain plays a regulatory role in the IgM pentamer-hexamer biosynthesis. It is interesting to note that hexameric IgM displays important biological advantages over pentamers in activation of complement [33, 34]. The fact that IgA and IgM, devoid of the J-chain, do not bind secretory component (SC) [35] suggests that pIgR/SC needs to bind not only to the Fc region of IgA, but also to the J-chain to form a stable IgA2–SC–J complex. The local humoral immune response is mainly mediated by secretory IgA, which plays a major role in protecting the mucosal surface against the invasion of pathogenic agents. SC present in the molecules of secretory IgA antibodies has a double role. First, it enhances the stability of the antibody by conferring resistance to the proteolytic attack of bacteria or local proteases [36], and second, it ensures, through its multiple carbohydrate residues, appropriate tissue localization by anchoring the antibody to mucus lining the epithelial surface [37]. Because of its crucial role, the J-chain is well conserved among different species, and the presence of a homologous peptide has been also found in the invertebrates. In the absence of J-chain, IgA is secreted as a monomer, the form most common in the blood. In humans, J-chain a is a polypeptide of 15 kDa containing eight cysteine residues of which six are involved in intrachain disulfide bridges and two in disulfide bridges with the *α* or *μ* chains. Furthermore, this polypeptide contains one site of N-glycosylation [30]. We found this protein to be present only in FRD patients, and this finding is noticeable considering that IgA deficiencies have been associated with chronic enteropathies in the dog [38], but also that no differences in J-chain encoding mRNA were found between healthy dogs and patients with chronic diarrhea [31]. Unfortunately, being the first study of that kind in the dog, it is difficult to define the reason behind J-chain presence in fecal samples from FRD dogs (and its absence in healthy controls). We can only speculate that this presence could be due to the rupture of mucosal immunocytes, followed the subsequent release of these polypeptides into the intestinal lumen (and their retrieval in fecal samples), or if it may represent a consequence of an increased immune system activation due to the dysbiosis likely associated with the condition, or even both. Actually, it is known that, besides B cells, J-chain expression has been detected in developing lymphocytes [39], dendritic cells (DCs) [40], and intestinal epithelial cells [41], none of which express secretory forms of Ig heavy chains. These studies point to novel, perhaps primordial, functions for J-chain; in particular J-chain also plays a role in the reduction of activation of complement. J-chain negative IgM hexamers are 15-20 times more effective at activating complement than J-chain positive IgM pentamers [29]. A consequence of this lack of complement activation it allows J-chain positive pIgM to bind antigens, as during intestinal dysbiosis, without causing excessive damage to enterocytes membranes through complement activation [42]. Since inflammation is generally suppressed in the intestinal epithelium, it is possible that this control somehow contributes to upregulation and secretion of J-chain.

With regard to the other proteins found in a significantly different amount in the two groups of dogs (Table 1), but almost all identified with a low score in MASCOT database, we believe that if confirmed in their nature as reported below they can be considered as presumable contaminants, i.e., spot I and L (species:* Streptomyces sp.*; accession number WP_ 046507073.1; MASCOT database score: 19 and a.n. WP_073935025.1; MASCOT database score 31, respectively) [43], as coming from feed or soil, i.e., spot A (species: Bos Taurus; a.n. gi|27819608; MASCOT database score 16), K (species:* Oryza sativa*; a.n. gi|50725157; SONAR database), K1 (species:* Zea mays*; a.n. ONM18096.1; MASCOT database score 18), S (species:* Microbulbifer donghaiensis*; a.n. WP_073272148.1; MASCOT database score 19), U (species:* Pontibacillus chungwhensis*; a.n. WP_036785728.1; MASCOT database score 33) [44, 45], as or being part of animals' microflora, i.e., spot ID T (species:* Staphylococcus aureus*; a.n. EZW68888.1; MASCOT database score 19) [46].

## 4. Conclusions

The most noticeable result of the present study is the finding of immunoglobulin J-chain isoform 1 in dogs suffering from FRD and its absence in control dogs. Being the first study of that kind in the dog it is unfortunately difficult to interpret these data, but we believe that this is a first important step in the study of fecal proteome in dogs suffering from chronic enteropathies, with possible important perspectives in diagnosis and monitoring these conditions; especially if considering the ease in obtaining samples to be analyzed. Certainly, the present results need to be confirmed by further studies and also it is important to underline that once identified biomarker/s by the 2DE analysis it would be desirable to develop a faster and simpler technique to allow its/their identification in the feces sample such as enzymatic activity or through the use of specific antibodies.

## Figures and Tables

**Table 1:** Identification of significantly changed fecal proteins from healthy dogs and dogs suffering from food responsive diarrhea (FRD).

Spot ID^a^	Mr. (kDa) / pI^b^ (±SD)	Normalized quantity **Healthy** (x10^3^)^b^	Normalized quantity **FRD** (x10^3^)^b^	Protein name^c^	Score	Species^c^	Sequences^c^	Mr. (kDa) / pI^c^	Accession number
A	12.4±0.4/7.9±0.2	550±61	179±21	Hemoglobin subunit beta	16^∗^	*Bos Taurus*	LLVVYPWTQR	15.9/7.01	gi|27819608

I	14.6±0.9/8.7±0.3	161±23	422±102	Hypothetical protein	19^∗^	*Streptomyces sp.*	PAAAAGTAVQ	14.1/9.5	WP_046507073.1

K	17.3±0.7/ 9.5±0	359 ± 130	58± 21	Putative Cytochrome P450	§	*Oryza sativa*	RTLVVSTAAAAAD LYR	54.8/ 9.1	gi|50725157

K1	17.1±0.2/9.8±0	0	126 ± 113	UDP-N-acetylglucosamine diphosphorylase	18^∗^	*Zea mays*	RIPSVHGYTSGLK	20.5/8.5	ONM18096.1

L	22.3±0.8/5.1±0.2	0	386±79	Isopentenyl-diphosphate delta-isomerase	31^∗^	*Streptomyces sp.*	QSGPRPFDPQEVA	21.1/5.3	WP_073935025.1

O1	15.0±0.7/4,7±0.2	0	118±17	not found	-				

O2	14.8±0.7/ 4.9±0.2	0	27.3±17.1	Immunoglobulin J chain isoform 1	§	*Canis lupus familiaris*	IIPSPDDPNE IVER	18.1/4.7	gi|57095596

O3	14.9±0.9/5.2±0.1	0	101±47	not found	-				

O4	13.7±0.6/5.6±0.1	0	113±32	not found	-				

S	11.4±0.4/5.7±0.1	36±19	94±46	DTW domain-containing protein	19^∗^	*Microbulbifer donghaiensis*	KTNTGALALAQCGNLVER	17.4/5.7	WP_073272148.1

T	9.9±0.8/6.2±0.1	60±25	197±95	hypothetical protein U973_01647	19^∗^	*Staphylococcus aureus*	IAKGLETAINAINE	10.6/5.8	EZW68888.1

U	44.8±1.3/5.3±0.2	148±41	528±155	Coproporphyrinogen III oxidase	33^∗^	*Pontibacillus chungwhensis*	EPVHYKEEMEEQ	44.6/5.6	WP_036785728.1

^a^Assigned spot ID as indicated in Figure 1.

^b^Experimental values calculated from the 2-DE maps by the PDQuest software.

^c^MASCOT (^∗^) and SONAR (^§^) results (SwissProt & NCBInr databases).

## Data Availability

The data used to support the findings of this study are included within the article.
